# Should scholar be the new interprofessional competency?

**Published:** 2019-11-28

**Authors:** Kerry Wilbur

**Affiliations:** 1 University of British Columbia, British Columbia, Canada

Interprofessional education (IPE) is widely endorsed as the gateway to enhanced collaborative patient care. When students from two or more professions learn about, from, and with one another in joint learning activities, interdisciplinary perceptions and attitudes improve and collaborative knowledge and skills increase.^1^ In turn, such early and structured immersion into overlapping and complementary patient care roles is intended to facilitate the shared decision-making these students can expect as part of teams in their future careers.^2^

Different frameworks exist to guide integration of IPE programming into health professional curricula worldwide.^3^ Most, more or less, hinge on the development of competencies deemed necessary for effective collaborative practice, such as communication and ethical values.The contribution of these skill sets to the positive and productive function of patient care teams is both apparent and universal. For example, abilities to listen and constructively consult, discuss, and debate are obvious elements for collegial interactions. Respecting patient dignity and maintaining confidentiality reflects a commitment to professional conduct and engenders trusting relationships. Such behaviours and attitudes are further recognized as collective competencies given they have been adopted by many North American health professional training programs as discipline-specific educational outcomes. By both accounts, these principles also apply to the scholar competency role.

The origins of a health professional as a scholar are rooted in the ability of physicians to practice evidence-based medicine (EBM).From 50 years agowhen McMaster University launched a novel medical program where students first learned about patient problems concurrent with epidemiology and biostatistics, Canadian physicians-in-training today are meant to ‘identify pertinent evidence, evaluate it using specific criteria, and apply it in their practice’ as part of the scholar competency role.^4^Similarly, the American Association of Medical Colleges outlines an entrustable professional activity milestone whereby trainees ‘form clinical questions and retrieve evidence to advance patient care’.^5^Albarqouni et al recently enlisted over 200 multidiscipline clinicians and academics from 28 countries to agree upon a set of evidence-based practice competencies for health professional teaching and learning.^6^ Through Delphi survey and consensus processes,they arrived at a set of 68 core competencies further organized into introductory concepts and five main evidence-based practicesteps including: ask, acquire, appraise and interpret, apply, and evaluate.While the authors acknowledge somecompetencies likely require modification to suit specific needs of any given discipline, most are indeed fundamental skills broadly applicable across professions. Examples include converting clinical questions into structured, answerable formats; constructing and carrying out an appropriate search strategy; distinguishing evidence-based from opinion-based clinical practice guidelines; explaining the evidence to patients and integrating their preferences into decision-making processes; and managing clinical decision-making uncertainty in practice. The consensus statement represents an important step towards unifying expectations of evidence-based practice. The details can guide instructional and assessment design and delivery in health professional curricula and for a number of programs, these may fall within the scholar competency role (or its counterparts like critical inquiry and evidence or evidence-informed patient care) (Table 1).

**Table 1:** Evidence-based practice competencies among Canadian health professional education frameworks

Nursing	Medicine	Occupational Therapy	Pharmacy	Physiotherapy	Respiratory Therapy
**Research, Methodologies, Critical Inquiry & Evidence:** The ability to seek, locate & interpret a broad range of Information knowledge, evidence, methodologies, and practice observations within the profession and across disciplines The ability to formulate research questions arising from nursing practice and analyze research findings	**Scholar:** Engage in the continuous enhancement of their professional activities through ongoing learning Integrate best available evidence into practice	**Thinks Critically:** Demonstrates effective and evidence-based problem solving and judgment to address client needs. **Engages in Professional Development:** Reviews various sources of information and new knowledge and determines applicability to practice Adapts to changes in practice using evidence, practice standards, and best practices	**Scholar:** Apply medication therapy expertise to optimize pharmacy care, pharmacy services and health care delivery Integrate best available evidence into pharmacy practice.	**Scholarship:** Use an evidence-informed approach in practice Engage in scholarly inquiry Maintain currency with developments relevant to area of practice	**Provide Evidence-informed, Patient-centred, Respiratory Care:** Apply evidence to practice **Demonstrate Critical Thinking and Reasoning Skills:** Analyze the data pertinent to the clinical situation in order to make a decision

If as Nandiwada&Kormos further contend, this work substantiates the existence and importance of evidence-based practice competencies across health professions, howdo they expressly promote shared or collaborative care?^7^ That is, how are these competencies truly interprofessional in nature and not simply common skills expected by many different professions? First, this set of competencies is an acknowledgement that a majority of professionals employ evidence-based practice in patient care. Although decision-making may occur within the discrete scope of a profession’s practice, we can be assured of a common language when this care is informed by reported literature, such as randomized controlled trials, meta-analyses, or clinical practice guidelines. Like the fundamental aspects of interprofessional communication (e.g. consultation, negotiation, respect, and active listening techniques) these competencies represent an opportunity for standardization of practice principles across patient care providers. Furthermore, when teams of various disciplines convene to develop treatment protocols or care pathways, there is a shared understanding of how the evidence under consideration may be interpreted.Clinicians may enlist similar approaches to navigate incomplete available data or ambiguous study findings to make treatment choices.

Interprofessional competencies are meant to promote collaborative care that ultimately improves patient outcomes. Integration of evidence-based competencies into shared decision-making clearly has the potential to do so. Patient adherence may be enhanced when care providers can clearly explain the data underpinning the rationale for the selected treatment and offer realistic estimates of potential risk. Clinicians who can interpret and synthesize a wide array of literature may have greater abilities to individualize care while also incorporating patient preferences. In this regard, optimal evidence-based practice must be enabled by the communication and collaboration skills endorsed by both profession-specific and interprofessional competency frameworks. Just as effective team-based care must draw upon the collective contributions of its diverse members, quality healthcare also relies on this complement of a clinician’s knowledge, skills, and behaviors.
